# Andean Tuber Ulluco (*Ullucus tuberosus*): Phenolic Profiling by UV-Vis Spectrophotometry and UHPLC-ESI-MS/MS

**DOI:** 10.3390/foods15050956

**Published:** 2026-03-09

**Authors:** Bianca Guzmán Condarco, Beatriz A. Vivanco Retamal, Catherine V. Tessini Ortiz

**Affiliations:** 1 Department of Chemistry, Universidad Técnica Federico Santa María, Av. España 1680, Valparaíso 2340000, Chile; bianca.guzman@sansano.usm.cl (B.G.C.); beatriz.vivanco@usm.cl (B.A.V.R.); 2 Faculty of Sciences, Universidad de Valparaíso, Blanco 951, Valparaíso 2340000, Chile

**Keywords:** Andean tubers, antioxidant activity, flavonoids, FRAP assay, phenolic compounds, phytochemical profile, UHPLC-ESI-MS/MS, ultrasonic-assisted extraction, *Ullucus tuberosus*

## Abstract

*Ullucus tuberosus* is an underutilized Andean tuber of South America, representing a highly nutritious food source valuable in bioactive compounds and used in traditional medicine by the local population. Despite its potential as a functional food, limited information is available regarding the phenolic composition of its diverse varieties, hindering its revalorization and potential biotechnological applications. In this study, the total phenolic content (TPC), antioxidant activity (AA), and thirteen individual phenolic compounds were investigated in five ulluco varieties using UV-Vis spectrophotometry and ultra-high-performance liquid chromatography, coupled with electrospray ionization and a triple quadrupole mass spectrometer (UHPLC-ESI-MS/MS). Statistical analyses revealed significant differences (*p* < 0.05) among the varieties in TPC, AA, and the concentration of the four quantified flavonoids. The major flavonoids found were rutin, quercetin-3-glucoside, kaempferol-3-rutinoside, and isorhamnetin-3-rutinoside, using solid-phase extraction (SPE-C8) as a cleanup step for ultrasound-assisted extracts, achieving satisfactory precision and recovery. Principal Component Analysis (PCA) effectively discriminated the samples based on their phenolic profiles, AA, and TPC. These findings contribute to the revalorization of ulluco varieties by providing new insights into their phenolic composition and demonstrating their potential as a source of health-promoting bioactive compounds.

## 1. Introduction

The ullluco (*Ullucus tuberosus*) is a highly nutritious food crop cultivated in the Andean region of South America, primarily in the central Andes, extending from Colombia to Bolivia [1,2,3,4]. This region is known for producing a wide variety of ulluco tubers that differ in colors (including purple, yellow, white, orange, green, or a combination of these), size (ranging from 2 to 15 cm), and shape (spherical, cylindrical, or ovoid) [5]. Unlike potatoes, ulluco has a thin peel that does not need to be removed for consumption and a mucilaginous center that contributes to its distinctive texture.

Beyond its nutritional value, ulluco has a long-held place in traditional Andean medicine, where it is used to alleviate stomach discomfort and to treat burns due to its skin-regenerative properties [6]. These attributes have generated increasing scientific interest in studying this tuber and have contributed to its reintroduction and commercialization in other countries although it remains an underutilized crop [7]. However, this situation has begun to change in recent years.

Recent studies have shown that Andean foods are rich in bioactive compounds, particularly phenolic compounds, and exhibit high antioxidant capacity [8,9]. Andean tubers, such as ulluco, are not only a source of essential nutrients but also contain bioactive compounds, including betalains and phenolic compounds [10,11,12,13,14,15]. Phenolic compounds—which include phenolic acids, flavonoids, lignans, tannins, and stilbenes—are secondary metabolites synthesized by plants that play a crucial role in food properties such as color, flavor, and oxidative stability [16,17]. Beyond their vital relevance for plant defense against pests, pathogens, and environmental stress, phenolic compounds are widely documented for their biological activities, including antioxidant, anti-inflammatory, antimutagenic, anticancer, anti-obesity, neuroprotective, and cardioprotective effects, thus contributing to the prevention of chronic and degenerative diseases [18,19,20,21,22].

The content and phytochemical diversity in *Ullucus tuberosus* may be influenced by multiple factors, including genotype, phenotype, climatic conditions, agronomic practices, and postharvest aspects such as storage and processing [23]. Therefore, ulluco varieties could exhibit significant differences in their phenolic composition and antioxidant activity, which can be assessed using spectrophotometric assays and high-resolution chromatographic techniques such as ultra-high-performance liquid chromatography, coupled with electrospray ionization and a triple quadrupole mass spectrometer (UHPLC-ESI-MS/MS). Recently, Pacheco et al. [12] have employed high-performance liquid chromatography (HPLC) coupled with a diode array detector (DAD) and mass spectrometry (MS) for the determination of phenolic compounds in the yellow-pink variety of ulluco. HPLC is generally preferred over gas chromatography (GC) because it does not require prior derivatization of phenolic compounds for their analysis. Despite the crop’s broad genetic diversity and the known presence of bioactive constituents, current knowledge of the chemical composition and phenolic profiles of different ulluco varieties remains limited, and comprehensive studies employing advanced analytical techniques remain scarce. Addressing this gap is essential to gain a deeper understanding of their phytochemical diversity and evaluate their potential as a functional food ingredient.

The extraction of phenolic compounds is a critical step in their analysis, since several factors, such as the extraction method, solvent type, extraction time, temperature, and sample particle size, influence the yield and efficiency of extraction. Ultrasound-assisted extraction (UAE) is an unconventional alternative that offers several advantages over traditional techniques, such as lower solvent consumption, minimal sample requirements, and shorter extraction times [24]. The fundamental principle of this technique is the acoustic cavitation, a phenomenon in which ultrasonic waves induce the formation, growth, and collapse of microbubbles [25]. This process generates microjets and localized regions of elevated temperature and pressure, which promote cell wall rupture and enhance mass transfer between the sample and the extraction solvent, thus improving the extraction efficiency [25,26]. To the best of our knowledge, no study has been reported that evaluates the relationships among different ulluco varieties based on their phenolic profiles using advanced analytical techniques and principal component analysis (PCA).

The objective of this study was to explore similarities and differences among five ulluco varieties available in local Chilean markets for human consumption, based on their phenolic profiling using UV-Vis spectrophotometry and UHPLC-ESI-MS/MS. The effects of ethanol concentration, extraction temperature, and sonication time on TPC and AA were evaluated for the UAE optimization. Furthermore, analytical parameters such as linear range, limit of detection (LOD), limit of quantification (LOQ), repeatability, intermediate precision, and recovery were assessed for the spectrophotometric and chromatographic methods. This study provides new insights into the phenolic profiling of ulluco varieties and highlights their potential as a source of health-promoting bioactive compounds.

## 2. Materials and Methods

### 2.1. Chemicals

Ethanol absolute (≥99.5%), sodium carbonate anhydrous (≥99.5%), iron (III) chloride (97%), 2,4,6-Tris (2-pyridyl)-s-triazine (≥98%), (±)-6-hydroxy-2,5,7,8-tetramethyl-chromane-2-carboxylic acid (≥97%), Folin & Ciocalteu’s phenol reagent 2N, gallic acid anhydrous (≥98%), 4-hydroxy-benzoic acid (≥98%), p-coumaric acid (≥98%), rutin (≥94%), quercetin dihydrate (≥98%), (-)-epicatechin (≥98%), and kaempferol (≥90%) were obtained from Sigma-Aldrich (Merck, St. Louis, MO, USA). Chlorogenic acid (≥98.75%) and ferulic acid were purchased from MP Biomedicals (Eschwege, Germany). Caffeic acid (≥98%) was obtained from ChromaDex (Longmont, CO, USA). Isorhamnetin-3-rutinoside (≥95%), kaempferol-3-rutinoside (≥95%), and quercetin-3-glucoside (≥95%) were provided by Phytolab (Vestenbergsgreuth, Germany). Acetonitrile grade LC/MS gradient (99.9%) and methanol grade LC/MS (99.9%) from Fisher Scientific (Waltham, MA, USA), deionized ultra-pure water (18.2 MΩ cm^−1^) was obtained from a Milli-Q system Millipore (Burlington, MA, USA).

### 2.2. Samples

**Figure 1:**
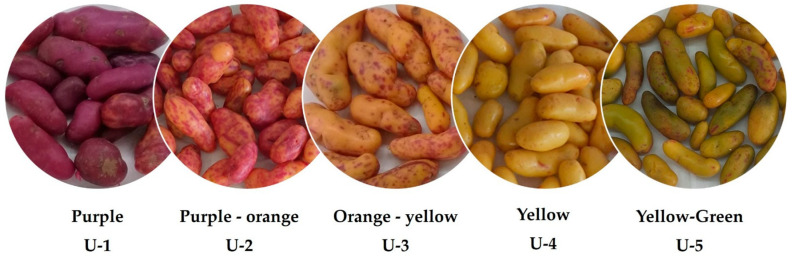
Ulluco tubers obtained in local Chilean markets.

Ulluco tubers were commercially obtained from the local market “La Vega”, located in the Metropolitan Region of Santiago, Chile. A total of 10 kg of samples was purchased from the same vendor on the same date to minimize variability. After acquisition, tubers were selected based on uniformity of color, absence of visible defects, and similar commercial maturity stage. The samples were gently cleaned using absorbent paper and a small amount of deionized water to remove soil residues. Figure 1 shows the five ulluco varieties collected. Subsequently, the tubers were lyophilized (peel and pulp combined), finely ground to obtain a homogeneous powder, and stored in a desiccator at room temperature in the dark until analysis.

### 2.3. Ultrasound-Assisted Extraction (UAE)

A unifactorial design was employed in ultrasound-assisted extraction to explore and evaluate the effects of ethanol concentration (10–90%, *v*/*v*), temperature (30–75 °C), and extraction time (10–50 min) on the extraction yield of phenolic compounds and their antioxidant activity in the purple ulluco sample. The selection of these variables and their respective ranges was based on previous studies focused on the extraction of phenolic compounds from food and plant matrices [24,27,28,29].

The extraction procedure was adapted from the method described by Xia et al. [30] with minor modifications. In brief, 0.2000 g of finely ground ulluco sample was weighed into a capped glass tube, to which 10 mL of ethanol at the selected concentration was added. The tube was sealed and placed in a bath sonicator (Model UC-30A, Biobase Scientific, Jinan, China) operating at a constant frequency of 50 Hz. After sonication, the extracts were centrifuged at 4000 rpm for 15 min at room temperature. An aliquot of 5 mL of the resulting supernatant was transferred to a 50 mL glass beaker and concentrated at 37 °C for 1 h in a thermostatic water bath. The resulting residue was then reconstituted with deionized water and quantitatively transferred to a 10 mL volumetric flask (diluted extract) for subsequent analysis.

### 2.4. Spectrophotometric Methods

The maximum absorbance wavelength of the colored complexes corresponding to total phenolic content (TPC) and antioxidant activity (AA) was determined using a UV–Vis spectrophotometer (UNICAM UV/VIS Spectrometer UV4, Unicam Instruments, Cambridge, UK) within the 400–800 nm range.

The extract obtained as described in Section 2.3 (final volume adjusted to 10 mL) was used as the stock solution for spectrophotometric determinations of total phenolic content (TPC) and antioxidant activity (AA). When necessary, additional dilutions were performed to ensure that absorbance values remained within the linear range of the respective calibration curves.

For the Folin–Ciocalteu assay, 1 mL of the appropriately diluted extract was used in the reaction mixture. For the FRAP assay, 3 mL of the stock extract was transferred to a 5 mL volumetric flask and adjusted to volume with 50% ethanol–water (*v*/*v*) to obtain a working solution. From this solution, 150 µL were used for the colorimetric reaction.

All measurements were performed using a 1 cm path-length quartz cuvette. For absorbance determination, 4 mL and 3 mL of the final reaction mixture were required for TPC and AA analyses, respectively.

#### 2.4.1. Folin–Ciocalteu Method for Total Phenolic Content Determination

The Folin–Ciocalteu method is widely used to assess total phenolic content (TPC) in various food matrices due to its simplicity and reproducibility. The reaction is based on an electron transfer mechanism, in which antioxidant compounds act as reducing agents and the Folin–Ciocalteu reagent functions as an oxidizing agent [31].

The reaction mixture was prepared by combining 1 mL of diluted extract, 2 mL of deionized water, and 1 mL of Folin–Ciocalteu reagent (10%, *v*/*v*) in a 10 mL volumetric flask. After 5 min, 1 mL of sodium carbonate solution (20%, *w*/*v*) was added, and the volume was adjusted to 10 mL with deionized water. The mixture was incubated in a thermostatic water bath at 25 °C for 1 h, and absorbance was measured at 700 nm.

TPC was expressed as mg gallic acid equivalents (GAE) per 100 g dry weight (DW).

#### 2.4.2. FRAP Method for Determination of Antioxidant Activity (AA)

The FRAP (Ferric Reducing Antioxidant Power) assay is based on the reduction of the Fe^3+^–TPTZ complex (ferric 2,4,6-tripyridyl-s-triazine) to the intensely blue Fe^2+^–TPTZ complex under acidic conditions. This reduction occurs through electron transfer from antioxidant compounds present in the sample. The assay was performed according to Thaipong et al. [32], with slight modifications. The FRAP reagent was freshly prepared by mixing 300 mM sodium acetate buffer (pH 3.6), 10 mM TPTZ solution in 40 mM hydrochloric acid, and 20 mM ferric chloride solution in a volumetric ratio of 10:1:1 (*v*/*v*/*v*).

For analysis, 150 µL of diluted extract or standard solution was mixed with 2.850 mL of FRAP reagent. The solution was incubated in a thermostatic water bath at 35 °C for 30 min, and absorbance was measured at 597 nm. A reagent blank was prepared and analyzed under identical conditions.

AA was expressed as mg Trolox equivalents (TE) per 100 g dry weight (DW).

#### 2.4.3. Analytical Parameters of the Spectrophotometric Methods

Analytical parameters, including linearity, limits of detection (LOD), limits of quantification (LOQ), precision, and accuracy, were evaluated following the guidelines established by the International Conference on Harmonization (ICH Q2(R1)) to ensure the reliability and quality of the analytical method [33]. Linearity was assessed using calibration curves constructed from eight concentration levels of gallic acid (10–140 mg/L) and Trolox (5–150 mg/L), prepared from stock standard solutions of 1000 mg/L and 500 mg/L, respectively. Blank solutions were prepared and measured identically for both spectrophotometric methods. The instrumental LOD and LOQ were calculated based on the residual standard error of the regression (*S*_y/x_) and the slope (b) of the calibration curve, using the equations LOD = 3.3 (*S*_y/x_)/b and LOQ = 10 (*S*_y/x_)/b.

The method’s precision and accuracy were evaluated using the purple ulluco sample under optimized extraction conditions. Precision was determined in terms of repeatability (intra-day, one analyst on the same day in one laboratory) and intermediate precision (inter-day, one analyst over three non-consecutive days in the same laboratory) by calculating the relative standard deviation (RSD). Accuracy was assessed through recovery experiments by spiking the purple ulluco sample with known concentrations of gallic acid at three levels (20, 36, and 65 mg/g) for the TPC method and Trolox at three levels (2, 8, 13 mg/g) for the FRAP method. Recoveries were calculated by the formula: Recovery (%) = [(*C*_f_ − *C*_u_)/*C*_a_] × 100, where *C*_u_ is the concentration of unspiked matrix (original concentration), *C*_f_ is the concentration of spiked matrix, and *C*_a_ is the concentration of added analyte.

### 2.5. Determination of Phenolic Compounds by UHPLC-ESI-MS/MS

#### 2.5.1. Instrument and Chromatographic Conditions

The determination of phenolic compounds was performed using a UHPLC-ESI-MS/MS system (UltiMate 3000 Series, Thermo Scientific, Waltham, MA, USA) equipped with a diode array detector (DAD) set at 210, 280, 320, and 360 nm, coupled to a triple quadrupole mass spectrometer (TSQ Fortis, Thermo Scientific, Waltham, MA, USA) with a heated electrospray ionization (H-ESI) source.

Chromatographic optimization was carried out using a standard mixture of thirteen phenolic compounds to achieve satisfactory resolution and peak shape. Separation was achieved on a Hypersil Gold™ C18 column (1.9 µm, 50 mm × 2.1 mm i.d., Thermo Scientific, Waltham, MA, USA) maintained at 25 °C. The mobile phase consisted of acetonitrile (Solvent A) and water containing 0.1% formic acid (Solvent B). The gradient elution program at a flow rate of 0.3 mL/min was as follows: 0–1 min, 95–90% B; 1–5 min, 90–88% B; 5–18 min, 88–87% B; 18–18.5 min, 87–60% B; 18.5–24.5 min, 60–95% B; 24.5–30 min, 95% B. The injection volume was 5 µL for both samples and standard solutions.

The mass spectrometer was operated in negative electrospray ionization mode. The optimized source parameters were as follows: spray voltage 2.5 kV; sheath gas 50 (arbitrary units); auxiliary gas 10 (arbitrary units); sweep gas 1 (arbitrary units); ion transfer tube temperature 300 °C; vaporizer temperature 350 °C. Q1 and Q3 were operated at a resolution of 1.2 FWHM. The collision-induced dissociation (CID) gas pressure was set to 1.5 mTorr. Preliminary experiments were conducted in full-scan mode within a mass range of 100–800 *m*/*z* to evaluate precursor ions and optimize MS/MS transitions. Quantification of the thirteen phenolic compounds was performed in Selected Reaction Monitoring (SRM) mode using optimized precursor-to-product ion transitions and collision energies.

Data acquisition and processing were carried out using Chromeleon™ 7 Software, Version 7.2 SR5 (Thermo Fisher Scientific Inc., Waltham, MA, USA).

**Figure 2:**
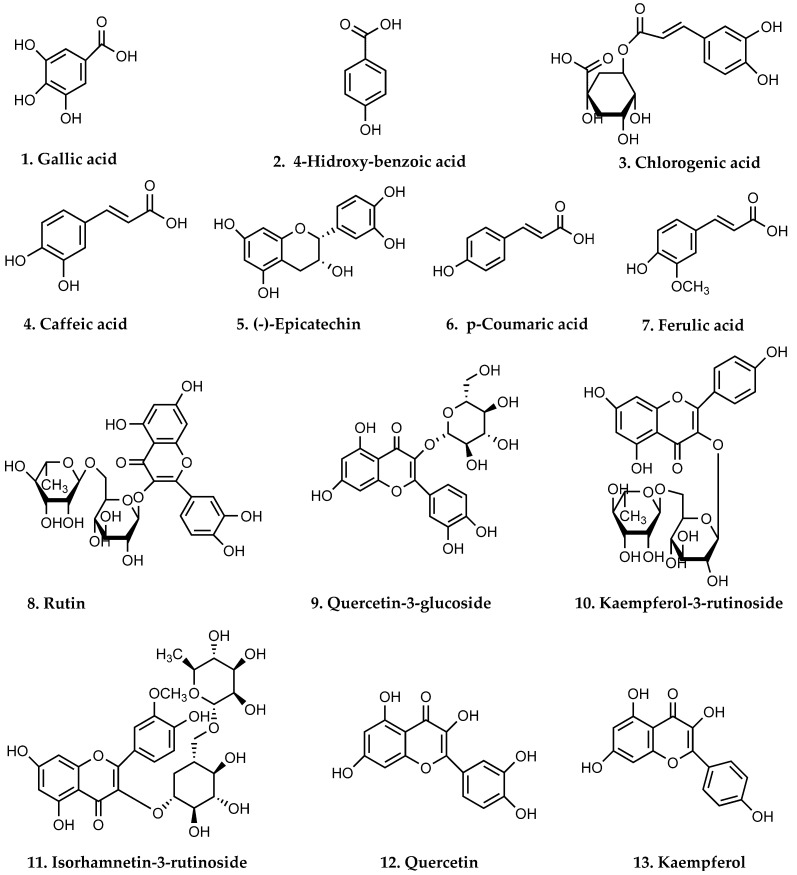
Chemical structures of 13 phenolic compounds investigated in five varieties of ulluco by UHPLC-ESI-MS/MS.

Figure 2 shows the chemical structures of the 13 phenolic compounds investigated in ulluco by UHPLC-ESI-MS/MS.

#### 2.5.2. Preparation of the Standard Solutions

Stock standard solutions of 1.00 mg/mL were prepared by dissolving each phenolic compound in methanol and kept in a refrigerator at −4 °C. Working standard solutions were prepared as needed by appropriate dilution of the analyte stock solutions in mobile phase ACN: 0.1% Formic acid in water (5:95, *v*/*v*). All standard solutions and samples were filtered through a syringe PTFE (0.22 μm, 13 mm, Jet Biofil, Guangzhou, China) membrane before injection into the UHPLC-ESI-MS/MS system.

#### 2.5.3. Clean up with Solid Phase Extraction (SPE)

The phenolic extracts obtained by UAE under optimized conditions were purified by solid-phase extraction (SPE) to reduce matrix interferences according to the procedure described by Pereira et al. [34], with minor modifications. The SPE cartridge (CEC08 Octyl, Endcapped, 500 mg, 3 mL; UCT, Bristol, PA, USA) was activated with 3 mL of methanol and conditioned with 3 mL of ultrapure water. A 2 mL aliquot of the phenolic extract was loaded onto the cartridge. The cartridge was washed with 5 mL of ultrapure water (non-acidified) to remove highly polar matrix components. Phenolic compounds were then eluted with 5 mL of methanol (≥99.8%, non-acidified). The eluate was evaporated to dryness under a gentle nitrogen stream at room temperature. The residue was reconstituted in 1 mL of mobile phase, centrifuged at 4000 rpm for 15 min at room temperature, and filtered prior to UHPLC-ESI-MS/MS analysis.

#### 2.5.4. Analytical Parameters of the Chromatographic Method

Linearity, LODs, LOQs, precision (Repeatability and intermediate precision), and accuracy were evaluated to detect and quantify the phenolic compounds by UHPLC-ESI-MS/MS. An external standard calibration approach was employed within the linear range, using fifteen mixed standard solutions containing thirteen phenolic compounds prepared from stock solutions at concentrations ranging from 1 to 3000 μg/L. Linear regression analysis was used to determine the slope, intercept, and determination coefficient of each calibration curve.

To determine the instrumental LODs and LOQs, mixed standard solutions at low concentrations (1 to 200 μg/L) were analyzed. Based on the calibration curve at low concentration for each phenolic compound, the LODs and LOQs were calculated as in Section 2.4.3. The LOQs were established as the lowest concentration at which the method was linear.

The repeatability of the method (UAE procedure, SPE-C8 cleanup, followed by chromatographic analysis) was determined using three independent extracts obtained from the U-1 sample. Each extract was injected in triplicate on the same day. Intermediate precision of the method was assessed by preparing extracts from the U-1 sample on three consecutive days, which were injected in triplicate in the chromatographic system. The variations were presented as RSD. Method accuracy was determined through recovery assays, in which a known amount of thirteen phenolic compounds was spiked into the purple ulluco sample at three concentration levels (40, 100, and 200 μg/g). Recoveries for each phenolic compound were calculated using the equation described in Section 2.4.3.

### 2.6. Statistical Analysis

The data were processed using Microsoft Excel and IBM SPSS Statistics (Version 21.0, IBM Corp., Armonk, NY, USA). Significant differences at the 95% confidence level (*p* < 0.05) were assessed by one-way analysis of variance (ANOVA), followed by Duncan’s multiple range test for mean comparison. All results are reported as mean ± standard deviation of three independent replicates. Pearson’s correlation analysis and Principal Component Analysis (PCA) were conducted in RStudio (version 2025.09.0+38; Posit Software, PBC, Boston, MA, USA) to evaluate the relationships among the measured variables and to explore similarity among the ulluco varieties. The PCA was performed using a data matrix consisting of five ulluco varieties and six analytical variables (TPC, FRAP, rutin, quercetin-3-glucoside, kaempferol-3-rutinoside, and isorhamnetin-3-rutinoside), where each variable corresponded to the mean value of three independent measurements.

## 3. Results

### 3.1. Ultrasonic-Assisted Extraction Method Optimization

**Figure 3:**
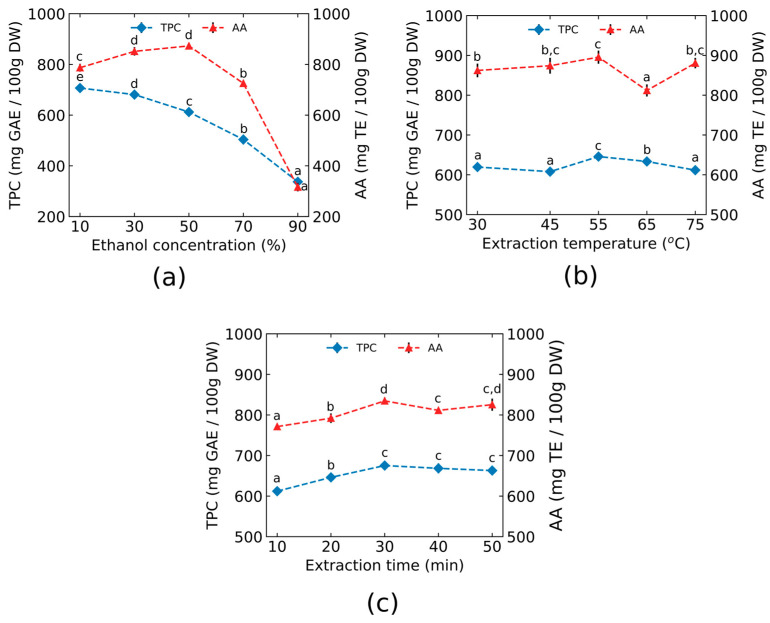
Single-factor experiments using ultrasonic-assisted extraction (UAE): (**a**) Effect of the ethanol concentration, (**b**) extraction temperature, and (**c**) extraction time on total phenolic content (TPC) and antioxidant activity (AA) in purple ulluco. Different letters within the same group denote statistically significant differences (*p* < 0.05) between means, as determined by Duncan’s multiple range test.

The unifactorial design enabled a systematic optimization of ultrasound-assisted extraction (UAE) in an underexplored food matrix such as *Ullucus tuberosus*. The effects of ethanol concentration, extraction temperature, and sonication time on total phenolic compounds (TPC) and antioxidant activity (AA) from purple ulluco are shown in Figure 3a–c. ANOVA results demonstrated that all three evaluated factors had a statistically significant effect (*p* < 0.05) on TPC and AA.

#### 3.1.1. Ethanol Concentration

Ethanol concentrations ranging from 10% to 90% (*v*/*v*) were evaluated at a fixed extraction temperature of 30 °C and a sonication time of 30 min. As shown in Figure 3a, the TPC decreased significantly with increasing ethanol concentration, yielding values between 337 ± 13 and 707 ± 8 mg GAE/100 g DW, whereas AA values ranged from 316 ± 17 to 873 ± 12 mg TE/100 g DW. The lowest yield values of TPC and AA were obtained with 90% ethanol. Based on these results, a 50% ethanol concentration was selected for subsequent extraction due to its phenolic yield (612 ± 6 mg GAE/100 g DW), highest antioxidant activity (873 ± 12 mg TE/100 g DW), and reduced co-extraction of highly polar interfering compounds.

#### 3.1.2. Temperature Extraction

The temperature is a critical factor in the extraction of phenolic compounds, as it reduces solvent viscosity, increases molecular movement rate, and enhances mass transfer, thereby improving the yield of UAE. The effect of temperature was assessed in the range from 30 °C to 75 °C using 50% ethanol and a constant sonication time of 30 min. As seen in Figure 3b, the TPC values ranged from 608 ± 6 to 646 ± 11 mg GAE/100 g DW, whereas the AA values ranged from 812 ± 15 to 896 ± 16 mg TE/100 g DW. The highest value of both TPC and AA was obtained at 55 °C. Therefore, 55 °C was chosen as the optimal extraction temperature.

#### 3.1.3. Time Extraction

Sonication time directly affects the efficiency and yield of phenolic compound extraction, as well as the resulting antioxidant activity [25]. In this extraction method, the effect of sonication time (10 to 50 min) on TPC and AA was evaluated using 50% ethanol at a fixed extraction temperature of 55 °C. As shown in Figure 3c, both TPC and AA values increased from 10 to 30 min of sonication, reaching maximum values at 30 min, namely, 675 ± 8 mg GAE/100 g and 835 ± 8 mg TE/100 g DW for TPC and AA, respectively.

Based on these results, the conditions of 50% ethanol: water (*v*/*v*), 55 °C, and 30 min of sonication were established as optimal conditions for extracting phenolic compounds with high antioxidant activity from ulluco tubers. Comparable conditions have been reported for similar matrices, such as potato by-products, using response surface methodology [35].

### 3.2. Spectrophotometric Determination of TPC and AA

#### 3.2.1. Analytical Parameters

**Figure 4:**
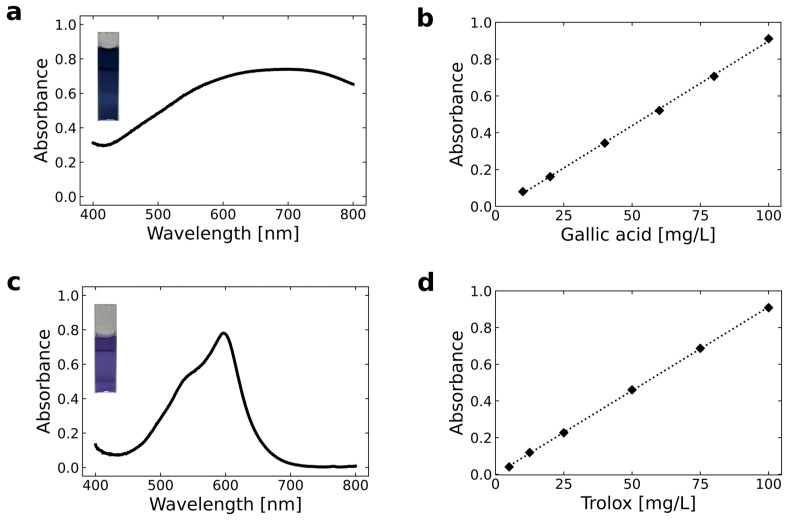
UV-Vis absorption spectra (400–800 nm) and corresponding calibration curves for the spectrophotometric methods: (**a**,**b**) Total phenolic content (TPC) using the Folin–Ciocalteu method and (**c**,**d**) antioxidant activity (AA) using the FRAP method.

Analytical parameters were evaluated to assess the performance of the UV–Vis spectrophotometric procedures employing the Folin–Ciocalteu reagent and the FRAP assay for the determination of TPC and AA, respectively. Figure 4 presents the corresponding absorption spectra and calibration curves.

A preliminary spectral scan (400–800 nm) was performed prior to wavelength selection. The maximum absorbance of the Folin–Ciocalteu complex was observed between 650 and 724 nm, and 700 nm was selected as the optimal wavelength under the established experimental conditions (Figure 4a).

This wavelength falls within the appropriate range (615–765 nm) reported for TPC determination. The optimal wavelength may vary depending on the reference compound, phenolic composition, sample matrix, instrumentation, and experimental conditions [36,37]. Several studies support the use of alternative wavelengths to the commonly reported 765 nm. For instance, TPC has been determined at 700 nm in mangosteen and grape seed extracts [38], at 650 nm in Lamiaceae species [39], and at 630 nm in citrus peels and other plant materials [40]. These findings highlight the importance of performing a preliminary wavelength scan for each specific matrix.

For the FRAP assay, the maximum absorbance of the Fe^2+^–TPTZ complex was observed at 597 nm (Figure 4c), which is consistent with the commonly reported value of 593 nm [41,42,43]. Calibration curves were constructed by plotting absorbance against concentration of gallic acid and Trolox standard solutions, as shown in Figure 4b,d.

Linearity, instrumental limits of detection (LOD) and quantification (LOQ), repeatability, intermediate precision, and recovery are summarized in Table 1. ANOVA confirmed that the calibration models were statistically significant (*p* < 0.05) over the concentration ranges of 10–100 mg/L (expressed as mg GAE) for TPC and 5–100 mg/L (expressed as mg TE) for AA. These ranges are consistent with those reported in previous studies employing gallic acid and Trolox standards for quantification [37,42,44,45].

The coefficients of determination (R^2^ > 0.9994) indicate excellent linearity across the studied range.

Repeatability and intermediate precision showed RSD values below 8%, which fall within the acceptable range (<10%) established for spectrophotometric methods [37].

Recovery experiments were performed at three concentration levels (low, medium, and high), each analyzed in triplicate (*n* = 9). Mean recovery values ranged from 109% to 112% for TPC and from 92% to 98% for FRAP, indicating satisfactory accuracy under the proposed experimental conditions. The slightly elevated recovery values observed for TPC are consistent with the well-documented nonspecific response of the Folin–Ciocalteu assay in complex plant matrices, where other reducing substances may contribute to the measured signal [46,47].

#### 3.2.2. Determination of TPC and AA in Five Ulluco Varieties

The spectrophotometric methods were applied to determine the TPC and AA in ulluco varieties under the optimized UAE conditions. The results obtained from Duncan’s test at *p* < 0.05 revealed statistically significant differences in the TPC and AA among the ulluco varieties. The TPC and AA results are summarized in Table 2. Yellow ulluco exhibited lower TPC and AA values compared to varieties showing mixed skin colors with varying pigment intensities. The antioxidant activity of the ulluco varieties followed the order: orange-yellow > purple-orange > purple > yellow > green-yellow.

### 3.3. UHPLC-ESI-MS/MS Method

#### 3.3.1. Simultaneous Separation and Detection of 13 Phenolic Compounds

The high selectivity and sensitivity of the UHPLC-ESI-MS/MS system enable the separation and detection of target analytes with satisfactory resolution within a relatively short analysis time. Achieving this requires careful optimization of chromatographic conditions and detector parameters (DAD and MS/MS) to prevent co-elution of structurally related analytes. The optimized chromatographic and mass spectrometric conditions are described in Section 2.5.1.

The mass scan range (100–800 *m*/*z*) was selected based on the available scientific literature, preliminary full-scan, and the expected precursor and product ions of the thirteen target phenolic compounds. During method optimization, extending the scan range beyond 800 *m*/*z* resulted in increased baseline noise and reduced analytical sensitivity. In contrast, restricting the scan range to 100–800 *m*/*z* improved the signal-to-noise ratio and enhanced detection sensitivity for the low-molecular-weight phenolic compounds investigated. Therefore, the selected mass range was analytically appropriate and optimized for the target analytes included in this study.

**Figure 5:**
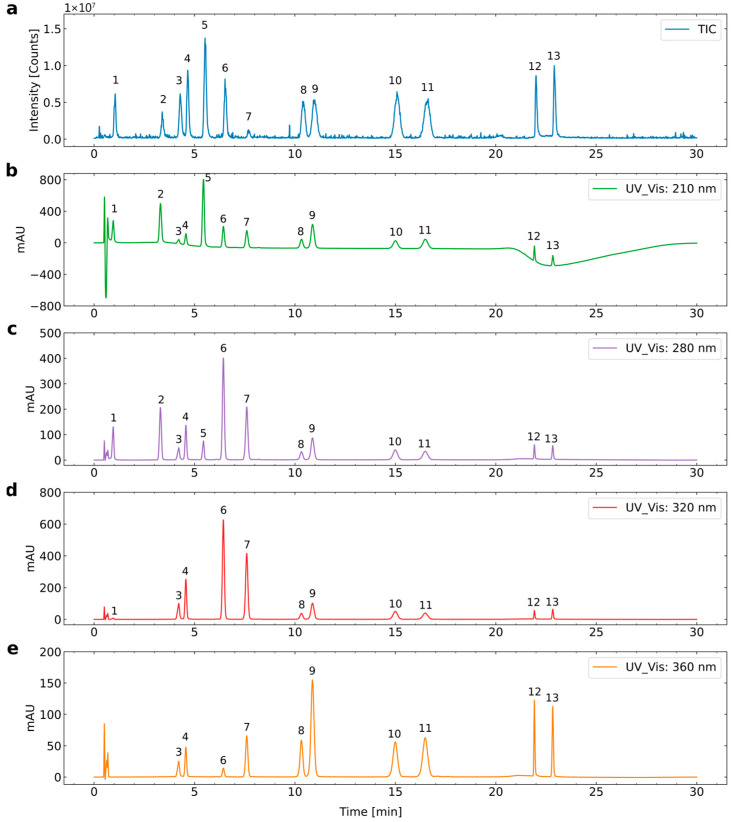
Simultaneous separation of 13 phenolic compounds by UHPLC-ESI-MS/MS in Scan Mode using a column Hypersil Gold ^TM^C18 (1.9 µm, 50 mm × 2.1 mm i.d Thermo Scientific, Waltham, MA, USA) (**a**) Total ion chromatogram (TIC): **1**. Gallic acid, **2**. 4-Hydroxy-benzoic, **3**. Chlorogenic acid, **4**. Caffeic acid, **5**. (-)-Epicatechin, **6**. p-Coumaric acid, **7**. Ferulic acid, **8**. Rutin, **9**. Quercetin-3-glucoside, **10**. Kaempferol-3-rutinoside, **11**. Isorhamnetin-3-rutinoside, **12**. Quercetin, **13**. Kaempferol; (**b**–**e**) chromatogram at 210 nm, 280 nm, 320 nm, and 360 nm, respectively.

Figure 5 shows the representative chromatograms of the simultaneous separation of 13 phenolic compounds investigated in ulluco by UHPLC-ESI-MS/MS in full scan mode.

The thirteen phenolic compounds investigated from ulluco varieties were (**1**) gallic acid, (**2**) 4-hydroxybenzoic acid, (**3**) chlorogenic acid, (**4**) caffeic acid, (**5**) (−)-epicatechin, (**6**) p-coumaric acid, (**7**) ferulic acid, (**8**) rutin, (**9**) quercetin-3-glucoside, (**10**) kaempferol-3-rutinoside, (**11**) isorhamnetin-3-rutinoside, (**12**) quercetin, and (**13**) kaempferol.

The mobile phase consisted of acetonitrile (Solvent A) and water containing 0.1% formic acid (Solvent B). The presence of formic acid in the aqueous phase contributed to stable and reproducible ionization conditions at the electrospray interface, while acetonitrile improved chromatographic resolution and minimized peak tailing. Adequate separation of all analytes was achieved, with resolution values (Rs > 1.5) confirming baseline separation. These conditions are consistent with previous reports demonstrating the effectiveness of this solvent system for phenolic compound analysis by UHPLC-DAD-ESI-MS/MS [48,49].

Each phenolic compound was identified based on its UV–Vis absorption spectrum, precursor ion ([M–H]^−^), specific MS/MS transition, retention time (tr), and comparison with authentic standard solutions and previously reported literature data. Selected Reaction Monitoring (SRM) mode was employed for the quantification of the thirteen phenolic compounds, using optimized precursor-to-product ion transitions and collision energies.

Compound **1** was identified as gallic acid based on its maximum absorption wavelengths (217.97 and 272.62 nm), transition [169.06/125.04, *m*/*z*], and retention time of 1.11 min. Similar wavelengths and MS/MS transitions for gallic acid have been reported in other studies [50,51].

Compound **2** was identified as 4-hydroxy-benzoic on its maximum wavelengths (197.96 and 256.69 nm), transition [137.09/92.81, *m*/*z*], and a retention time of 3.50 min. Compound **3** was identified as chlorogenic acid on its maximum absorption wavelengths (228.13 and 326.67 nm), transition [353.15/190.71, *m*/*z*], and retention time of 4.45 min. Also, the [353.5/191.3] transitions for chlorogenic acid were reported by Truong et al. [52]. Then, compound **4** was identified as caffeic acid on its maximum absorption wavelengths (228.08, 238,28, and 324.06 nm), transition [179.08/134.79, *m*/*z*], and a retention time of 4.82 min.

Compound **10** was identified as kaempferol-3-rutinoside on its maximum absorption wavelengths (267.77 and 346.33 nm), transition [593.21/284.55, *m*/*z*], and a retention time of 15.59 min. Similar transitions for the compounds **9** [463.08878/301, *m*/*z*] and **10** [593.15199/285, *m*/*z*] were found using a UHPLC-FT-ICR/MS [53].

Compound **11** was identified as isorhamnetin-3-rutinoside on its maximum absorption wavelengths (256.82 and 353.38 nm), transition [623.26/314.67, *m*/*z*], and a retention time of 17.01 min. Compound **12** was identified as quercetin on its maximum absorption wavelengths (246.07 and 376.26 nm), transition [301.09/272.03, *m*/*z*], and a retention time of 22.17 min.

Compound **13** was identified as kaempferol on its maximum absorption wavelengths (249.80, 264.34, and 375.31 nm), transition [285.08/255.19, *m*/*z*], and a retention time of 22.95 min. The MS/MS transitions of phenolic compounds **1**, **3**, **4**, **5**, **7**, **8**, **12**, and **13** align with the results reported by Yuan et al. [54].

Table 3 summarizes the optimized SRM conditions for the determination of thirteen phenolic compounds by UHPLC-ESI-MS/MS.

#### 3.3.2. Analytical Parameters of the Chromatographic Method

The results for linearity, the coefficient of determination, LODs, and LOQs are summarized in Table 4. The calibration curves exhibited good linearity, with all coefficients of determination ranging from 0.9974 to 0.9995. They were determined by plotting the peak area signal of the SRM mode against seven concentration levels of each mixed standard solution.

The instrumental LODs and LOQs of the analytes ranged from 1 to 65 µg/L and from 5 to 198 µg/L, respectively. Some values of LODs and LOQs obtained from the standard error of the calibration curve regression were comparable to those reported by Nzekoue et al. [55] and Cortese et al. [56], using HPLC-MS/MS. In general, the LOD and LOQ values achieved in this study were lower than those previously reported by Irakli et al. [57] and Wang et al. [58], demonstrating the high sensitivity of the proposed analytical method for the identification and quantification of 13 phenolic compounds.

Several studies have reported the use of SPE-C18 cartridges for the determination of phenolic compounds in food matrices [12,59,60,61]. In the present study, however, a C8 cartridge was selected for two main reasons. First, to remove matrix interferences from ulluco extracts and to protect the chromatographic system, which does not include a guard column and is therefore more susceptible to contamination and column overloading. To assess this effect, an aliquot of the U-1 extract was injected directly, without prior SPE-C8 cleanup. As shown in Supplementary Figure S1, both the TIC and UV-VIS chromatograms at 280 nm obtained after SPE-C8 treatment revealed a substantial reduction in interfering compounds, resulting in improved selectivity and sensitivity.

Second, Pacheco et al. [12] reported flavonols as the predominant phenolic group in yellow-pink ulluco. In agreement with this observation, the chromatograms of the U-1 extract (Supplementary Figure S1) displayed analytical signals at retention times corresponding to the flavonoids investigated. This suggests that the SPE-C8 treatment is suitable for chromatographic analysis. Subsequently, precision and recovery of the method were evaluated.

Table 5 summarizes the precision and recovery results obtained for four phenolic compounds in the purple ulluco sample.

The RSD values for repeatability and intermediate precision were <10%, demonstrating that the analytical method (including EAU method and UHPLC-ESI-MS/MS analysis) exhibited satisfactory precision.

Recovery values ranged from 86% to 98% for compounds **8**, **9**, **10**, and **11**, which were considerably higher than those observed for the remaining phenolic compounds, whose recovery percentages were notably low (below 36%). According to Pereira, O.R. et al. [34], recovery values between 50% and 120% are considered acceptable when accompanied by RSD values below 15%.

#### 3.3.3. Determination of Phenolic Compounds in Ulluco Varieties

**Figure 6:**
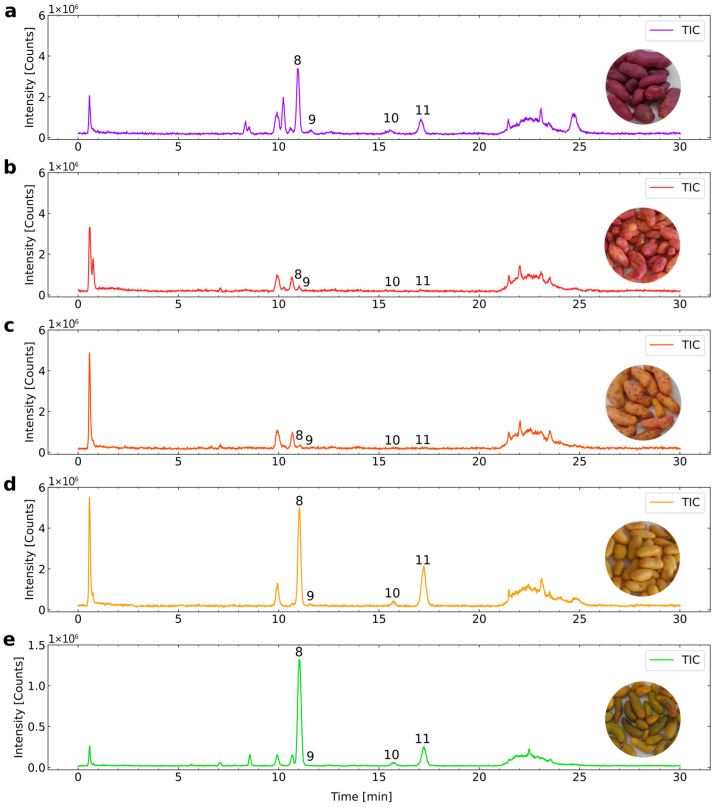
Total ion chromatograms (TICs) obtained by UHPLC-ESI-MS/MS for five varieties of *Ullucus tuberosus*: (**a**) purple (U-1), (**b**) purple–orange (U-2), (**c**) orange–yellow (U-3), (**d**) yellow (U-4), and (**e**) yellow–green (U-5). Peaks **8**. Rutin, **9**. Quercetin-3-glucoside, **10**. Kaempferol-3-rutinoside, **11**. Isorhamnetin-3-rutinoside.

To determine the phenolic composition of ulluco, the extracts obtained using the proposed method were analyzed by UHPLC-ESI-MS/MS to generate the chromatographic profiles for the five varieties. Total ion chromatograms (TICs) were examined to confirm compound presence, and the TICs of each ulluco variety are presented in Figure 6.

Flavonoids represented the most abundant group of phenolic compounds across all analyzed ulluco varieties in this work. The TICs clearly revealed the presence of analytes **8**, **9**, **10**, and **11** in the five samples. Among them, the purple, yellow, and yellow-green varieties exhibited more intense signals for rutin than the purple-orange and orange-yellow varieties. Compounds **1**–**7** were not detected in any variety within the 0–10 min retention window.

Based on the chromatograms recorded at 210, 280, 320, and 370 nm, four flavonoids—rutin, quercetin-3-glucoside, kaempferol-3-rutinoside, and isorhamnetin-3-rutinoside—were identified by comparing their UV–Vis spectra, deprotonated molecular ions ([M–H]^−^), and retention times with those of commercial analytical standards. Supplementary Figure S2 shows a representative chromatogram at 280 nm. Although additional signals appeared at the retention times of gallic acid, chlorogenic acid, caffeic acid, and ferulic acid, their mass spectra showed multiple molecular ions, indicating co-elution and low analyte abundance.

Quantification was subsequently performed in SRM mode, an unequivocal and highly selective approach for the identification and quantification of low-abundance analytes that could not be detected in the total ion chromatogram, and the results are reported in Table 6. The results revealed the presence of gallic acid in the purple, purple-orange, and orange-yellow varieties. In contrast, 4-hydroxybenzoic acid, (−)-epicatechin, and p-coumaric acid were not detected in all samples. Caffeic acid was identified in the purple, purple-orange, orange-yellow, and yellow-green varieties. Ferulic acid was detected in the purple-orange variety. Quercetin was detected only in the purple variety, and kaempferol in the orange-yellow variety.

Gallic acid was tentatively quantified in the purple, purple-orange, and orange-yellow varieties. In contrast, 4-hydroxybenzoic acid, (−)-epicatechin, and p-coumaric acid were not detected in all samples. Caffeic acid was identified in the purple, purple-orange, and orange-yellow varieties. Ferulic acid was detected only in the purple-orange variety. Quercetin was identified in the purple variety, and kaempferol in the orange-yellow variety.

The lowest values of rutin, quercetin-3-rutinoside, kaempferol-3-rutinoside and isorhamnetin-3-rutinoside were found in the orange-yellow variety. Quercetin 3-glucoside was identified and quantified in all ulluco varieties, with the highest concentration recorded in the purple variety. This flavonoid has not been reported for ulluco varieties so far.

According to ANOVA followed by Duncan’s test results, significant differences (*p* < 0.05) were observed among the varieties in the contents of rutin, quercetin-3-glucoside, kaempferol-3-rutinoside, and isorhamnetin-3-rutinoside. Statistical analysis was not conducted for compounds **1**, **2**, **3**, **4**, **5**, **6**, **7**, **12**, and **13** due to low recovery values and limited quantitative reliability.

### 3.4. Statistical Correlation and Principal Component Analysis (PCA)

**Figure 7:**
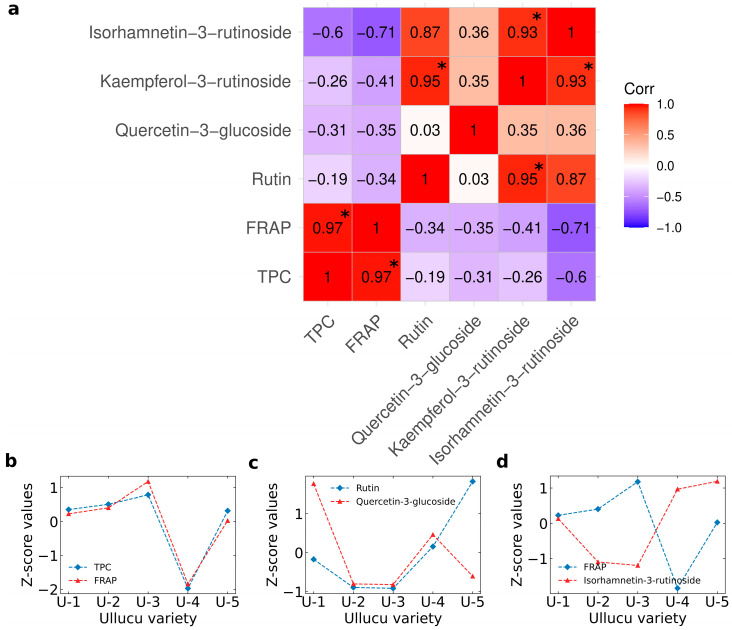
(**a**) Pearson’s statistical correlation (*p* < 0.05) between TPC, AA, rutin, quercetin-3-glucoside, kaempferol-3-rutinoside, and isorhamnetin-3-rutinoside from *Ullucus tuberosus* varieties; (*) statistically significant; (**b**) shows a positive correlation between TPC and FRAP; (**c**) shows a close-to-zero correlation between rutin and quercetin-3-glucoside, and (**d**) shows an anticorrelation between FRAP and Isorhamnetin-3-rutinoside.

Statistical correlation evaluates the strength and direction of the relation between two variables. A high positive correlation suggests that the variables are closely related and likely measure the same characteristic. Conversely, low correlations indicate that the variables may represent different characteristics. Statistical correlation among TPC, FRAP, rutin, quercetin-3-glucoside, kaempferol-3-rutinoside, and isorhamnetin-3-rutinoside from ulluco varieties is illustrated in Figure 7. The *p*-values of Pearson’s statistical correlation are shown in Supplementary Material Table S1.

A strong and statistically significant correlation (r = 0.97, *p* < 0.05) was observed between TPC and FRAP (Figure 7b), and between rutin and kaempferol-3-rutinoside (r = 0.95, *p* < 0.05). Kaempferol-3-rutinoside also showed a strong and significant correlation with isorhamnetin-3-rutinoside (r = 0.93, *p* < 0.05), suggesting that both compounds share similar structural and functional characteristics. As glycosylated flavonoids, they likely exhibit comparable antioxidant properties, which may contribute synergistically to the overall antioxidant capacity of the tubers.

In contrast, both TPC and FRAP showed negative correlations with rutin, quercetin-3-glucoside, kaempferol-3-rutinoside, and isorhamnetin-3-rutinoside, with the strongest negative correlation observed between FRAP and isorhamnetin-3-rutinoside (r = −0.71, *p* ≥ 0.05) (Figure 7d). This suggests that antioxidant activity could be associated with a structure-activity relationship, particularly kaempferol derivatives, in ulluco extracts. On the other hand, no significant correlation was observed between quercetin-3-glucoside and rutin (Figure 7c), suggesting that these compounds exhibit distinct properties and contribute independently to the overall antioxidant activity.

**Figure 8:**
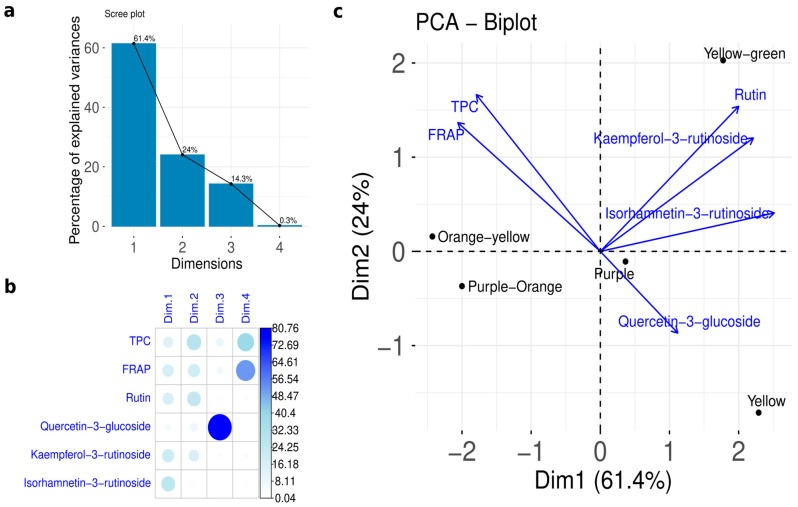
Principal component analysis (PCA) of ulluco varieties: (**a**) scree plot showing the percentage of variance explained by each principal component; (**b**) correlation matrix illustrating the relationships between TPC, FRAP, rutin, quercetin-3-glucoside, kaempferol-3-rutinoside, and isorhamnetin-3-rutinoside with the principal components, where circle size indicates the magnitude of the correlation; (**c**) PCA biplot showing the distribution of ulluco varieties and the contribution of the analyzed variables.

Subsequently, principal component analysis (PCA) was applied with the TPC, FRAP antioxidant activity, rutin, quercetin-3-glucoside, kaempferol-3-rutinoside, and isorhamnetin-3-rutinoside to explore the variability within the ulluco tubers. Figure 8 illustrates the PCA biplot for ulluco varieties.

The first and second components of the PCA explained 61.4% and 24% of the variance, respectively. That means that 85.4% of the variability in the experimental dates of TPC, FRAP antioxidant activity, rutin, quercetin-3-glucoside, kaempferol-3-rutinoside, and isorhamnetin-3-rutinoside is explained by the biplot of PCA. Isorhamnetin-3-rutinoside was the principal PC1 contributor (26.43%) followed by kaempferol-3-rutinoside (20.46%).

On the other hand, the principal contributor of PC2 was the TPC (29.54%), followed by rutin (25.30%) and FRAP antioxidant activity (19.93%). Also, it was noticed that three were well categorized around the origin. All flavonoids were positioned on the right side of the F1 axis in the PCA biplot, suggesting consistent bioactive properties associated with the composition of this group of phenolic compounds in green-yellow, purple, and yellow varieties (Figure 7b), indicating that TPC plays a major role in the antioxidant capacity. Furthermore, the coloration of the tubers may be associated with their antioxidant activity; the yellow ulluco variety exhibited lower TPC and FRAP values than the other varieties analyzed.

## 4. Discussion

### 4.1. Effect of Extraction Conditions on Phenolic Yield and Antioxidant Activity

The extraction efficiency of phenolic compounds from ulluco was influenced by ethanol concentration, extraction temperature, and sonication time. The use of hydroalcoholic mixtures favored the recovery of phenolic compounds with medium and high polarity, such as phenolic acids, flavonoids, and anthocyanins, which exhibit antioxidant activity and are more soluble in aqueous solvents. The presence of water modifies the polarity of ethanol, thereby enhancing its extraction efficiency [25,26,62]. These findings are consistent with previous reports indicating that ethanol concentrations between 50% and 60% are optimal for phenolic extraction from other food sources [35,63,64,65].

Temperature also played a relevant role in phenolic recovery and antioxidant activity. At temperatures above the optimum value, a decline in both total phenolic content (TPC) and antioxidant activity (AA) was observed, possibly due to thermal degradation of thermolabile phenolic compounds. Similar temperatures have been reported in other studies on food matrices as optimal for extracting phenolic compounds [37,38].

Sonication time directly affected the extraction efficiency. Beyond 30 min, a decline in both TPC and AA was observed, likely due to the degradation of thermolabile phenolic compounds caused by prolonged ultrasound exposure or heat accumulation. These observations are consistent with previous reports indicating that sonication times between 20 and 30 min yield optimal extraction results in food matrices [66,67,68].

### 4.2. Spectrophotometric Determination of TPC and AA and Varietal Differences

The spectrophotometric determination of TPC and AA revealed statistically significant differences among the ulluco varieties. These differences may be influenced by variations in phenolic composition among the samples.

Yellow ulluco exhibited lower TPC and AA values compared to varieties displaying mixed skin colors with varying pigment intensities. According to the results (Table 2), higher TPC and AA values were generally observed in more intensely pigmented varieties, particularly those ranging from orange to purple. This trend suggests a possible association between pigmentation intensity and phenolic composition, although color alone cannot be considered a direct indicator of antioxidant capacity. Muñoz et al. [69] previously reported that red and yellow ulluco peels exhibited higher FRAP antioxidant activity and TPC than their corresponding pulps, further supporting the influence of phenolic distribution within plant tissues.

The TPC values obtained in this study were higher than those reported by Pacheco et al. [4] for pink–yellow ulluco and other food matrices, such as purple sweet potato and white carrot. Differences in extraction solvent, temperature, time, and matrix characteristics may partly explain these discrepancies. Similarly, Pérez-Balladares et al. [70] reported lower TPC and antioxidant activity values for ulluco cultivated in Ecuador, obtained using methanolic extraction and expressed on a fresh weight basis. Nevertheless, these studies also highlight the relatively high antioxidant potential of ulluco compared to other plant-based foods.

### 4.3. Phenolic Profiling of Ulluco by UHPLC-ESI-MS/MS

The UHPLC-ESI-MS/MS analysis enabled the identification and quantification of individual phenolic compounds in ulluco varieties, providing detailed information on their phenolic composition. Flavonoids were identified as the predominant phenolic group in the studied five ulluco varieties, with rutin, quercetin-3-glucoside, kaempferol-3-rutinoside, and isorhamnetin-3-rutinoside consistently detected. Shamsudin, N.F. et al., [71] describe that flavonoids exhibit greater antioxidant activity due to several structural features, such as the number and position of hydroxyl groups, the presence of a double bond between carbon atoms 2 and 3, and a carbonyl group at position 4 in the C-ring. Additionally, their ability to interact synergistically with other antioxidants further enhances their overall antioxidant potential.

Previous studies have also reported the phenolic content of other ulluco varieties using chromatographic techniques. Pacheco et al. [12] tentatively determined non-anthocyanin compounds in Andean tubers by HPLC–DAD–ESI/MS^n^. In their study, they found that the yellow–pink variety of melloco or ulluco also contained flavonoids **8**, **10**, and **11**, although in lower concentrations compared with the five varieties analyzed in our research. Sánchez-Portillo et al. [14] identified hydroxycinnamic acids, methoxyflavonols, and other flavonols in both the flour and peel of red–purple variety by RP-HPLC-ESI-MS. Similarly, Zavaleta et al. [72] quantified flavonoids and phenolic acids using HPLC–UV/Vis detection at 370 nm and reported the presence of chlorogenic acid, ferulic acid, and rutin in ulluco samples. Moreover, the rutin content reported in their study is comparable to the levels observed in the present work for the purple and yellow varieties. More recently, Suárez-Calle et al. [73] identified gallic acid in white *Ullucus tuberosus* flour using HPLC–UV/Vis detection at 272 nm. These studies provide additional evidence supporting the potential of ulluco as a functional food due to its bioactive compounds and antioxidant capacity.

On the other hand, sample cleanup using SPE-C8 cartridges was effective in reducing matrix interferences and improving selectivity and sensitivity. Although SPE-C18 cartridges are commonly used for phenolic analysis, the use of a C8 cartridge was suitable for the chromatographic analysis of ulluco extracts, particularly for four flavonoids.

Regarding quantification, satisfactory precision and recovery were obtained for flavonoids, whereas phenolic and hydroxycinnamic acids showed lower recovery values. This behavior may be attributed to their higher polarity and partial elution during the water-washing step designed to remove highly polar matrix interferences. Consequently, the quantification of phenolic acids should be considered tentative, while the results obtained for flavonoids can be regarded as reliable.

### 4.4. Statistical Correlation and Principal Component Analysis

Statistical correlation analysis (see Section 3.4) revealed a strong and significant correlation between TPC and FRAP antioxidant activity, indicating that phenolic compounds play a major role in the antioxidant capacity of ulluco extracts. Similar relationships between phenolic content and antioxidant activity have been reported for other Andean tubers [74].

The coloration of the tubers may also be associated with their antioxidant activity [75], as yellow ulluco exhibited lower TPC and FRAP values than the purple, purple–orange, and orange–yellow varieties. This trend may be related to the presence of pigmented phenolic compounds such as anthocyanins, flavonoids, and other polyphenols. However, within the scope of this study, a direct causal relationship between tuber color and TPC/AA cannot be conclusively established. Ulluco samples may also contain other pigmented compounds, such as betalains, which have been previously reported in these tubers [10]. As noted by Muñoz et al. [69], antioxidant activity cannot be explained solely by total phenolic content; characterization of individual phenolic compounds is necessary to establish structure–activity relationships. In addition, sample processing and storage conditions may influence both pigmentation and antioxidant measurements.

Strong correlations were also observed among certain flavonoids, such as rutin and kaempferol-3-rutinoside, which may be attributed to their structural similarity. In contrast, negative correlations between TPC and FRAP with some individual flavonoids suggest that antioxidant activity cannot be explained exclusively by total phenolic content and may depend on structure–activity relationships, as previously reported [69].

Principal component analysis (PCA) effectively differentiated ulluco varieties based on their phenolic composition, antioxidant activity, and total phenolic content. The elevated levels of quercetin-3-glucoside in purple and yellow ulluco contributed to the separation of the purple–orange variety from these samples. Likewise, higher TPC and FRAP values contributed to the differentiation of the orange–yellow variety. Consequently, orange–yellow and purple–orange were positioned on the negative side of the PC1 axis in the PCA biplot. Overall, PCA revealed distinct clustering patterns reflecting variations in phenolic composition and antioxidant activity among the analyzed varieties.

### 4.5. Health Benefits of Phenolic Compounds Present in Ulluco Tubers

**Figure 9:**
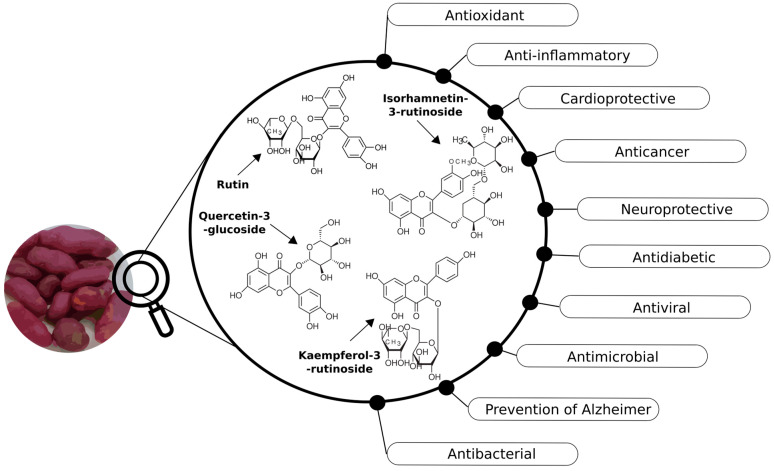
Schematic representation of pharmacological properties previously reported in the literature for rutin, quercetin-3-glucoside, kaempferol-3-rutinoside, and isorhamnetin-3-rutinoside.

Phenolic compounds have been widely reported to contribute to human health through antioxidant and anti-inflammatory mechanisms [76]. Figure 9 summarizes biological activities previously reported in the literature for phenolic compounds identified in ulluco tubers.

The flavonoids identified in ulluco, including rutin, quercetin-3-glucoside, kaempferol-3-rutinoside, and isorhamnetin-3-rutinoside, have been associated in the literature with various biological activities [77,78,79]. Rutinoside derivatives of isorhamnetin possess pharmacological properties associated with the prevention of obesity and tumor development, as well as cardioprotective, neuroprotective, and antioxidant activities. Rutinoside derivatives of kaempferol, such as kaempferol-3-rutinoside, display potent antioxidant, anti-inflammatory, cardioprotective, and antidepressant effects [79].

Phenolic acids detected in ulluco, such as gallic and chlorogenic acids, have also been reported to exhibit antimicrobial, antioxidant, anti-inflammatory, and antiviral activities [80,81].

These findings suggest that ulluco tubers may represent a valuable source of phenolic compounds for dietary intake and potential applications in food and biotechnology.

## 5. Conclusions

To our knowledge, this is the first study to report the phenolic profiling of *Ullucus tuberosus* varieties using UHPLC-ESI-MS/MS and UV-Vis spectrophotometry. Among the samples analyzed, the yellow ulluco extract exhibited the lowest phenolic content and antioxidant activity. Rutin, quercetin-3-glucoside, kaempferol-3-rutinoside, and isorhamnetin-3-rutinoside were quantified with satisfactory precision and recovery. Notably, quercetin-3-glucoside is reported here for the first time in ulluco tubers. Principal Component Analysis (PCA) proved to be an effective tool for exploring similarities and differences among the samples, providing a comprehensive understanding of the distribution and associations of phenolic compounds across the five varieties.

Overall, this work contributes to the valorization of this underutilized Andean crop by providing new insights into its phenolic composition and highlighting its potential as a source of bioactive compounds beneficial to health. Further studies employing advanced analytical techniques are needed to deepen the understanding of the functional properties of different ulluco varieties. In addition, future research could explore the integration of nondestructive approaches, such as machine vision systems, to evaluate external color attributes and their possible relationship with phenolic composition and antioxidant activity.

## Figures and Tables

**Table 1:** Analytical parameters of the spectrophotometric methods for the determination of total phenolic content (TPC) and antioxidant activity (AA) in ulluco.

Analytical Parameters	Folin–Ciocalteu Method	FRAP Method
Range lineal (mg/L)	LOQ − 100	LOQ − 100
Calibration equations	y = 0.0096x − 0.0183	y = 0.0091x + 0.0002
R^2^	0.9994	0.9998
LOD (mg/L)	3	2
LOQ (mg/L)	10	5
Repeatability (RSD, *n* = 9)	2%	2%
Intermediate Precision (RSD, *n* = 9)	4%	8%
% Recovery (*n* = 3)		
Low	112%	92%
medium	109%	98%
Hight	110%	98%

Calibration curves were prepared by diluting stock solutions of Gallic acid and Trolox to obtain different concentrations for TPC and AA, respectively; LOD: Limit of detection; LOQ: Limit of quantification; R^2^: coefficient of determination. Precision is expressed through repeatability (one analyst on the same day in one laboratory) and intermediate precision (one analyst on different days in the same laboratory). Accuracy is expressed through recovery by spiking the purple ulluco sample with known amounts of gallic acid and Trolox at three levels (20, 36, and 65 mg/g) and (2, 8, and 13 mg/g), respectively. RSD: Relative Standard Deviation.

**Table 2:** Total phenolic content and antioxidant activity in five *Ullucus tuberosus* varieties.

Sample	Ulluco Varieties	TPC Mean ± *s*	AA Mean ± *s*
U-1	Purple	681 ± 9 ^d^	992 ± 6 ^c^
U-2	Purple-orange	625 ± 5 ^b^	1017 ± 21 ^d^
U-3	Orange-yellow	645 ± 13 ^c^	1126 ± 9 ^e^
U-4	Yellow	456 ± 8 ^a^	698 ± 9 ^a^
U-5	Yellow-green	612 ± 13 ^b^	963 ± 8 ^b^

TPC: Total phenolic content obtained by the Folin–Ciocalteu method is expressed as mg gallic acid equivalents/100 g DW; AA: Antioxidant activity obtained by the FRAP method is expressed as mg of Trolox equivalents/100 g DW; *s*: Standard deviation; Values with different superscripts within the same column are statistically significantly different at *p* < 0.05 according to Duncan test.

**Table 3:** Optimized SRM conditions for the determination of phenolic compounds by UHPLC- ESI-MS/MS.

Analyte	MF	MW	t_r_ (min)	UV λ _max_ (nm)	Precursor Ion (*m*/*z*) [M-H]^−^	C. E. (V)	Product Ions (*m*/*z*)
**1**	C_7_H_6_O_5_	170.12	1.11	217.97, 272.62	169.06	15	**[125.04]**, 168.8
**2**	C_7_H_6_O_3_	138.12	3.50	197.96, 256.69	137.09	15	**[92.81]**, 93.54, 136.99
**3**	C_16_H_18_O_9_	354.31	4.45	228.13, 326.67	353.15	15	**[190.71]**, 191.46
**4**	C_9_H_8_O_4_	180.16	4.82	228.08, 238.28	179.08	15	**[134.79]**, 135.41
**5**	C_15_H_14_O_6_	290.27	5.84	206.08, 230.75	289.13	15	**[244.86]**, 288.85, 203.72, 289.57
**6**	C_9_H_8_O_3_	164.16	6.81	311.46, 229.83	163.10	15	**[119.10]**, 163.39
**7**	C_10_H_10_O_4_	194.18	8.03	324.05, 240.36	193.36	15	**[133.86]**, 177.63, 134.63
**8**	C_27_H_30_O_16_	610.52	10.99	258.30, 353.08	609.21	35	**[300.37]**, 299.56, 301.39
**9**	C_21_H_20_O_12_	464.38	11.47	353.08, 258.55	463.16	25	**[300.46]**, 299.59, 301.31
**10**	C_27_H_30_O_15_	594.52	15.59	267.77, 346.33	593.21	30	**[284.55]**, 285.36, 592.58, 283.57, 593.49
**11**	C_28_H_32_O_16_	624.55	17.01	256.82, 353.38	623.26	30	**[314.67]**, 315.42
**12**	C_15_H_10_O_7_	302.33	22.17	246.07, 376.26	301.09	25	**[272.63]**, 300.76, 273.46, 150.71, 255.46, 179.18
**13**	C_15_H_10_O_6_	286.23	22.95	249.80, 264.34	285.08	40	**[255.19]**, 181.94, 182.62, 227.51, 142.96, 93.35

Phenolic compounds: **1.** Gallic acid, **2.** 4-Hydroxy-benzoic, **3.** Chlorogenic acid, **4.** Caffeic acid, **5.** (-)-Epicatechin, **6.** p-Coumaric acid, **7.** Ferulic acid, **8.** Rutin, **9.** Quercetin-3-glucoside, **10.** Kaempferol-3-rutinoside, **11.** Isorhamnetin-3-rutinoside, **12.** Quercetin, **13.** Kaempferol, MF: Molecular Formule; MW: Molecular weight; V: Voltaje. Product ions correspond to the selected SRM transitions used for quantification. Bold values indicate the product ion used for quantification, while the remaining ions were used for confirmation.

**Table 4:** Linearity, LODs, and LOQs of 13 phenolic compounds by UHPLC-ESI-MS/MS in SRM mode.

N°	Phenolic Compounds	Lineal Range, µg/L	Regression Equations	R^2^	LOD, µg/L	LOQ, µg/L
**1**	Gallic acid	50–3000	y = 0.3909x – 23.439	0.9974	17	50
**2**	4-Hidroxy-benzoic acid	43–3000	y = 0.3217x – 0.3426	0.9982	14	43
**3**	Chlorogenic acid	7–3000	y = 4.6266x – 308.97	0.9983	2	7
**4**	Caffeic acid	26–3000	y = 2.9119x − 99.085	0.9978	9	26
**5**	(-)-Epicatechin	6–3000	y = 2.0048x + 9.236	0.9993	2	6
**6**	p-Coumaric acid	15–1000	y = 1.2108x + 1.4161	0.9987	5	15
**7**	Ferulic acid	8–1000	y = 0.1266x − 2.7177	0.9979	3	8
**8**	Rutin	9–3000	y = 10.384x − 199.12	0.9994	3	9
**9**	Quercetin-3-glucoside	11–3000	y = 21.791x − 383.01	0.9980	4	11
**10**	Kaempferol-3-rutinoside	17–3000	y = 11.479x − 46.996	0.9991	6	17
**11**	Isorhamnetin-3-rutinoside	25–3000	y = 11.838x + 230.19	0.9995	8	25
**12**	Quercetin	198–3000	y = 0.1931x − 22.305	0.9978	65	198
**13**	Kaempferol	82–3000	y = 0.1332x − 2.8757	0.9981	27	82

R^2^: coefficient of determination; LOD: Limit of detection; LOQ: Limit of quantification.

**Table 5:** Precision and recovery of phenolic compounds by UHPLC-ESI-MS/MS in SRM mode in purple ulluco.

N°	Phenolic Compounds	Repeatability (*n* = 9)		Intermediate Precision (*n* = 9)		Recovery (*n* = 3)		
						40 μg/g	100 μg/g	200 μg/g
		Mean	RSD	Mean	RSD	%	%	%
**8**	Rutin	162	6%	153	7%	98	94	86
**9**	Quercetin-3-glucoside	7.1	5%	7.6	7%	89	91	91
**10**	Kaempferol-3-rutinoside	28	5%	27	8%	91	94	92
**11**	Isorhamnetin-3-rutinoside	83	7%	85	10%	86	90	87

Method precision was evaluated using extracts obtained from the purple ullluco according to the procedure previously described for UAE and SPE-C8, and is expressed through repeatability (one analyst on the same day in one laboratory) and intermediate precision (one analyst on three consecutive days in the same laboratory); Results are expressed in μg/g in dry weight; Accuracy is expressed through recovery by spiking the purple ulluco sample with known amounts of 13 phenolic compounds at three levels (40, 100, and 200 µg/g). RSD: Relative Standard Deviation.

**Table 6:** The contents (µg/g) of 13 investigated phenolic compounds in *Ullucus tuberosus* varieties by UHPLC-ESI-MS/MS.

N°	Phenolic Compounds	U-1	U-2	U-3	U-4	U-5
		Mean ± *s*	Mean ± *s*	Mean ± *s*	Mean ± *s*	Mean ± *s*
**1**	* Gallic acid	3.2 ± 0.02	4.5 ± 0.1	4.0 ± 0.1	ND	ND
**2**	* 4-Hidroxy-benzoic acid	ND	ND	ND	ND	ND
**3**	* Chlorogenic acid	3.123 ± 0.002	3.07 ± 0.01	ND	ND	ND
**4**	* Caffeic acid	<LOQ	<LOQ	<LOQ	ND	ND
**5**	* (-)-Epicatechin	ND	ND	ND	ND	ND
**6**	* p-Coumaric acid	ND	ND	ND	ND	ND
**7**	* Ferulic acid	ND	˂LOQ	ND	ND	ND
**8**	Rutin	143 ± 3 ^b^	14.1 ± 0.6 ^a^	10.0 ± 0.2 ^a^	201 ± 7 ^c^	499 ± 17 ^d^
**9**	Quercetin-3-glucoside	7.3 ± 0.5 ^d^	0.92 ± 0.01 ^a^	0.882 ± 0.003 ^a^	4.1 ± 0.2 ^c^	1.41 ± 0.03 ^b^
**10**	Kaempferol-3-rutinoside	29 ± 1 ^b^	6.1 ± 0.3 ^a^	8.0 ± 0.2 ^a^	27 ± 1 ^b^	45 ± 2 ^c^
**11**	Isorhamnetin-3-rutinoside	81 ± 6 ^b^	9.3 ± 0.2 ^a^	3.6 ± 0.1 ^a^	129 ± 4 ^b^	142 ± 5 ^c^
**12**	* Quercetin	˂LOQ	ND	ND	ND	ND
**13**	* Kaempferol	ND	ND	˂LOQ	ND	ND

The results are expressed in µg/g DW (Dry Weight); (*): Tentative quantification; U-1: Purple—ulluco; U-2: Purple-orange; U-3: Orange-yellow; U-4: Yellow; U-5: Yellow-green; LOQ: Limit of quantification; *s*: Standard Deviation; ND: Not Detected. Different letters (a–d) within a row indicate statistically significant differences (*p* < 0.05) among the different varieties.

## Data Availability

The original contributions presented in the study are included in the article/Supplementary Material; further inquiries can be directed to the corresponding author upon reasonable request.

## Supplementary Materials

The following supporting information can be downloaded at: https://www.mdpi.com/article/10.3390/foods15050956/s1, Figure S1. Chromatograms of purple ulluco extract by UHPLC-ESI-MS/MS: (a) total ion chromatogram (TIC) with and without SPE-C8; (b) UV–Vis chromatogram at 280 nm with and without SPE-C8. Figure S2. UV–Vis chromatogram at 280 nm of five varieties of *Ullucus tuberosus* using UHPLC-ESI-MS/MS: (a) purple, (b) purple–orange, (c) orange–yellow, (d) yellow, and (e) yellow–green. Peaks: 8, rutin; 9, quercetin-3-glucoside; 10, kaempferol-3-rutinoside; 11, isorhamnetin-3-rutinoside. Table S1. *p*-Values of Pearson’s statistical correlation.

## Abbreviations

The following abbreviations are used in this manuscript:

UAE	Ultrasonic assisted-extraction
TPC	Total phenolic content
AA	Antioxidant Activity
LOD	Limit of detection
LOQ	Limit of quantification
FRAP	Ferric reducing antioxidant
UHPLC	Ultra-High-performance liquid chromatography
GC	Gas chromatography
DAD	Diode array detector
RSD	Relative standard deviation
*s*	Standard deviation
SPE	Solid-phase extraction
H-ESI	High-electro spray ionization
GAE	Gallic acid equivalent
TE	Trolox equivalent
PCA	Principal component analysis
MS	Mass spectrometry
