# Supplementary material for: Andean Tuber Ulluco (Ullucus tuberosus): Phenolic Profiling by UV-Vis Spectrophotometry and UHPLC-ESI-MS/MS
Source: Foods. 2026 Mar 9;15(5):956. doi: 10.3390/foods15050956 (PMC12984910; doi:10.3390/foods15050956)
Supplement: Supplementary file 1 [file foods-15-00956-s001.zip › foods-4151518-supplementary.pdf]

By Guzmán Condarco et al. **Supplementary material**

Figure S1

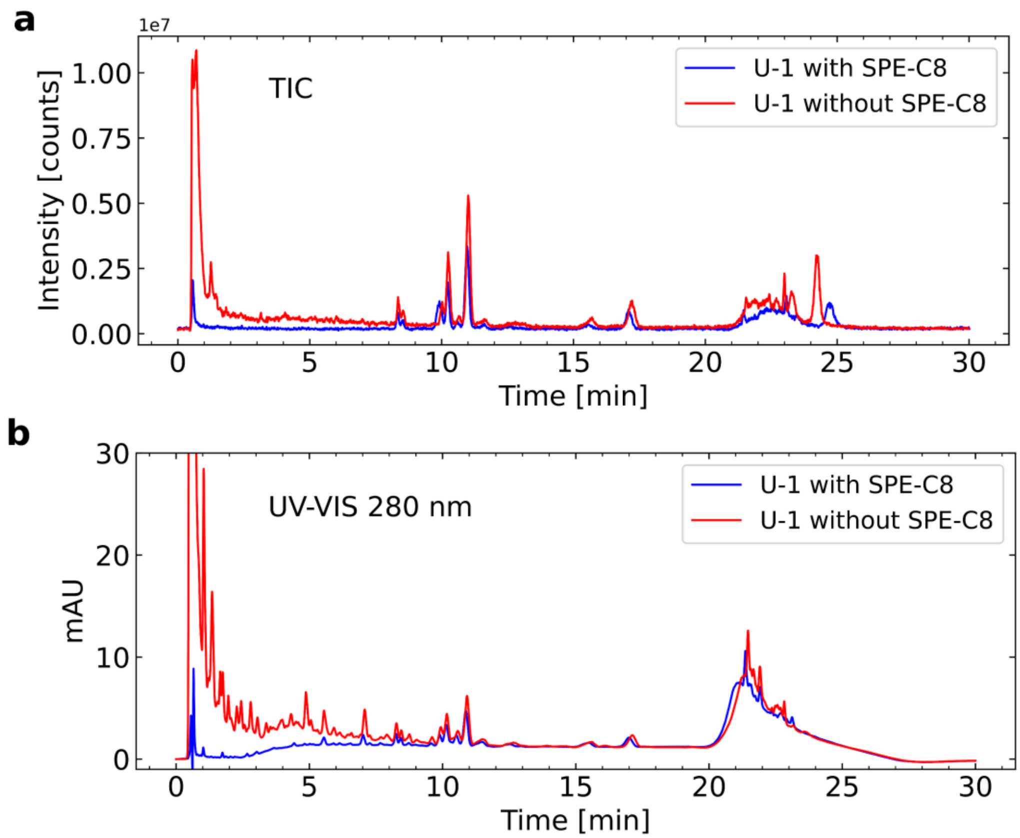

Figure S1. Chromatograms of purple ulluco extract by UHPLC-ESI-MS/MS: (a) total ion chromatogram (TIC) with and without SPE-C8; (b) UV-Vis chromatogram at 280 nm with and without SPE-C8.

Figure S2

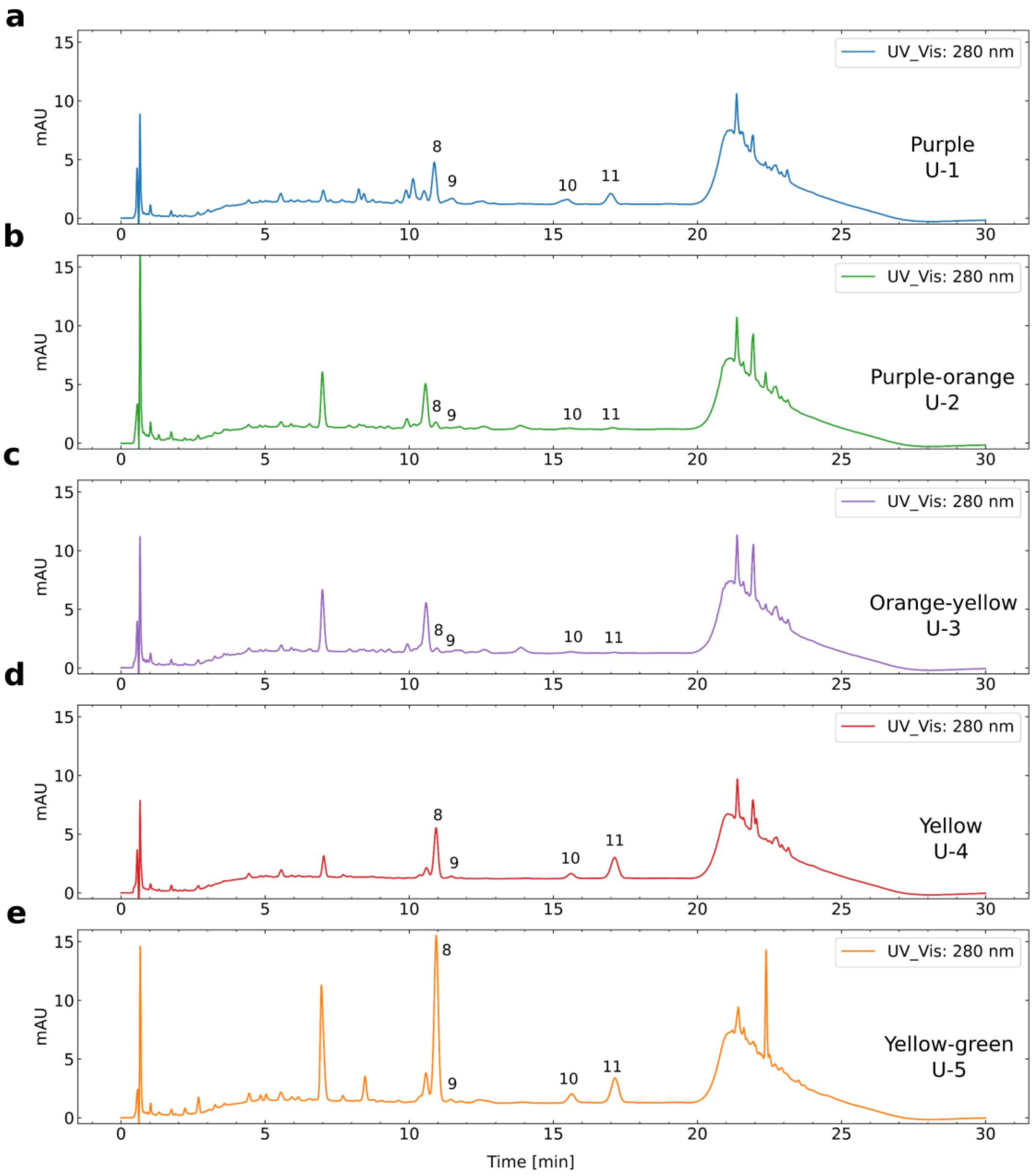

Figure S2. UV-Vis chromatogram at 280 nm of five varieties of *Ullucus tuberosus* using UHPLC-ESI-MS/MS: (a) purple, (b) purple—orange, (c) orange—yellow, (d) yellow, and (e) yellow—green. Peaks **8**. Rutin, **9**. Quercetin-3-glucoside, **10**. Kaempferol-3-rutinoside, **11**. Isorhamnetin-3-rutinoside.

Table S1

Table S1. *p*-Values of Pearson´s statistical correlation.

	TPC	FRAP	Rutin	Quercetin-3-glucoside	Kaempferol-3-rutinoside	Isorhamnetin-3-rutinoside
TPC	NA	0.006*	0.756	0.616	0.668	0.289
FRAP	0.006*	NA	0.572	0.563	0.490	0.175
Rutin	0.756	0.572	NA	0.966	0.015*	0.053
Quercetin-3-glucoside	0.616	0.563	0.966	NA	0.568	0.547
Kaempferol-3-rutinoside	0.668	0.490	0.015*	0.568	NA	0.022*
Isorhamnetin-3-rutinoside	0.289	0.175	0.053	0.547	0.022*	NA

(*): Significantly at $p<0.05$
